# Variations in the Obesity Gene “*LEPR*” Contribute to Risk of Type 2 Diabetes Mellitus: Evidence from a Meta-Analysis

**DOI:** 10.1155/2016/5412084

**Published:** 2016-04-18

**Authors:** Ming Ming Yang, Jun Wang, Jiao Jie Fan, Tsz Kin Ng, Dian Jun Sun, Xin Guo, Yan Teng, Yan-Bo Li

**Affiliations:** ^1^Eye Hospital, The First Affiliated Hospital of Harbin Medical University, Harbin 150001, China; ^2^The Centre for Endemic Disease Control, Chinese Center for Disease Control and Prevention, Harbin Medical University, Harbin 150001, China; ^3^Department of Ophthalmology and Visual Sciences, The Chinese University of Hong Kong, Sha Tin 999077, Hong Kong; ^4^Department of Endocrinology, The First Affiliated Hospital of Harbin Medical University, Harbin 150001, China

## Abstract

Leptin is a hormone protein regulating food intake and energy expenditure. A number of studies have evaluated the genetic effect of leptin (*LEP*) and leptin receptor (*LEPR*) genes on T2DM. This study aimed to investigate the association between these gene polymorphisms and T2DM by a systematic review and meta-analysis. Published studies were identified through extensive search in PubMed and EMBASE. A total of 5143 T2DM cases and 5021 controls from 14 articles were included in this study. Five functional variants in* LEPR* were well evaluated. Meta-analysis showed that rs1137101 (p.R223Q) was significantly associated with T2DM in all genetic models: allele model (OR = 1.27, 95% confidence interval (CI) = 1.13–1.42), dominant model (OR = 1.19, 95% CI = 1.05–1.35), homozygote model (OR = 1.82, 95% CI = 1.38–2.39), and recessive model (OR = 1.75, 95% CI = 1.35–2.28), with minimal heterogeneity and no indication of publication bias. Similar associations with T2DM were also found for rs62589000 (p.P1019P) and 3′UTR ins/del, although the data was obtained from a small number of studies. For the other two polymorphisms rs1137100 (p.R109K) and rs8179183 (p.K656N), they were not significantly associated with T2DM. Our results provide robust evidences for the genetic association of rs1137101 (p.R223Q) in* LEPR* with T2DM susceptibility.

## 1. Introduction

Type 2 diabetes mellitus (T2DM) is a group of metabolic disorders with insulin secretion deficiency or insulin resistance (IR) and characterized by hyperglycemia. The prevalence of diabetes increases significantly in recent decades, affecting about 6% of adult population globally. Therefore, it is one of the major health care challenges in the world [1]. T2DM is a heterogeneous and polygenic disease associated with increased risk of several complications, such as cardiovascular disease, ischemic heart disease, and diabetic retinopathy [2]. Over the past decades, great achievements have been made in clinical diagnosis and interventions as well as elucidating the underlying pathogenesis of diabetes. Obesity, food intake, and energy expenditure have long been recognized as important key factors in the etiology of diabetes [3]. Besides, genetic factors also play a pivotal role in the development of diabetes and identified multiple T2DM-associated genes, providing additional insights into the disease mechanisms [4].

Leptin is a hormone protein important in regulating food intake and energy expenditure for energy balance, fertility, and metabolism, which are mediated by the cell surface leptin receptor (LepR) [5, 6]. It is suggested that there is a connection between energy metabolism and obesity [7]. Under normal condition, leptin can reduce appetite and increase sympathetic activity. Notably, leptin is also known to facilitate glucose utilization and improves insulin sensitivity [8, 9]. Apart from its role in obesity, recent studies have drawn attention to the role of leptin in the pathogenesis of T2DM and insulin resistance.

LepR is encoded by the leptin receptor gene (*LERP*) on chromosome 1p31. Several functional variants with possible biological effects on metabolism regulation have been extensively investigated for the genetic predispositions on diabetes and its complications. These gene polymorphisms include rs1137101 (p.Arg223Gln or p.R223Q), rs1137100 (p.Arg109Lys or p.R109K), rs8179183 (p.Lys656Asn or p.K656N), rs62589000 (p.Pro1019Pro or p.P1019P), 3′-untranslated region (UTR) ins/del, rs1805134 (p.Ser343Ser or p.S343S), and rs2228301 (p.Asn567Asn or p.N567N) [10–23].

Although many studies have elucidated the association between T2DM and* LEPR* gene, the conclusion is still controversial because of small sample size in each study and lack of robust replication. We therefore conducted a systematic review and meta-analysis to evaluate the genetic impacts of* LEPR* gene polymorphisms on the risk of T2DM.

## 2. Methods

### 2.1. Searching Strategy and Inclusion Criteria

A comprehensive literature search was conducted in online databases, MEDLINE (Medical Literature Analysis and Retrieval System Online) and EMBASE (via Ovid) engines up to July 2015. The following medical subject headings and keywords were used for search strategy: “leptin”, “leptin receptor”, “LEP”, “LEPR”, “gene(s)”, “polymorphism(s)”, “mutation(s)”, “variant(s)”, “diabetes”, “diabetic”, “DM”, and “diabetes mellitus”. References lists of the retrieved articles and reviews were also screened for additional articles not captured by electronic search. Eligible studies were defined as the following criteria: (1) case-control study; (2) investigating the association between T2DM and* LEPR* polymorphisms; (3) sufficient genotype distribution data in case and control groups; (4) study samples being unrelated individuals drawn from clearly defined populations; (5) written in English. The exclusion criteria were defined as studies on animals, case reports, reviews, abstracts, editorial comments, and reports with incomplete data.

### 2.2. Data Extraction and Quality Appraisal

The following information was extracted from each study: first author, year of publication, country of studies, ethnicity, sample size, age, gender, allele/genotypic frequencies, and body mass index (BMI). If the test for Hardy-Weinberg equilibrium (HWE) was not reported, it was tested by the genotype data. The quality of studies was evaluated independently by two investigators (M. M. Yang and J. Wang) according to the Newcastle-Ottawa Scale (NOS). Uncertainties were resolved by discussions or by consensus with a third reviewer (J. J. Fan). NOS evaluated studies with a star-rating system ranging from 0 (lowest) to 9 (highest) stars, which was based on three study components, including selection, comparability, and outcome assessment. Studies with more than 5 points were evaluated as qualified.

### 2.3. Statistical Analysis

Pooled odds ratios (ORs) and corresponding 95% confidence intervals (CI) were used to estimate the association strength between* LEPR* polymorphisms and T2DM risk. The regression coefficients and the associated standard errors (SE, 95% CI) were combined using meta-analytic software. The combined ORs were, respectively, calculated by four genetic models (allele, dominant, recessive, and homozygous). HWE among controls was evaluated by *χ* ^2^ test and *P* < 0.05 was considered as significant disequilibrium. Both Cochran's *Q* statistic testing degree of heterogeneity across studies and the index *I* ^2^ statistic quantifying the proportion of heterogeneity between studies were calculated; *P* value less than 0.10 for the *Q*-test and *I* ^2^ above 50% were considered as statistically significant. If there was significant heterogeneity, the random effects model would be used to analyze the pooled ORs; otherwise, the fixed effects model would be applied. We used univariate random effects metaregression to investigate the potential sources of heterogeneity, such as, ethnicity, sample size, source of control, and publication years. Sensitivity analysis was used to assess the stability of results by systematically removing each study and reassessing the significance, whereas funnel plot and Egger's test were used to assess the potential publication bias. Statistical analyses were conducted by Review Manager (version 5.3, Copenhagen: The Nordic Cochrane Centre, The Cochrane Collaboration, 2014).

## 3. Results

### 3.1. General Characteristics of the Included Studies

**Figure 1:**
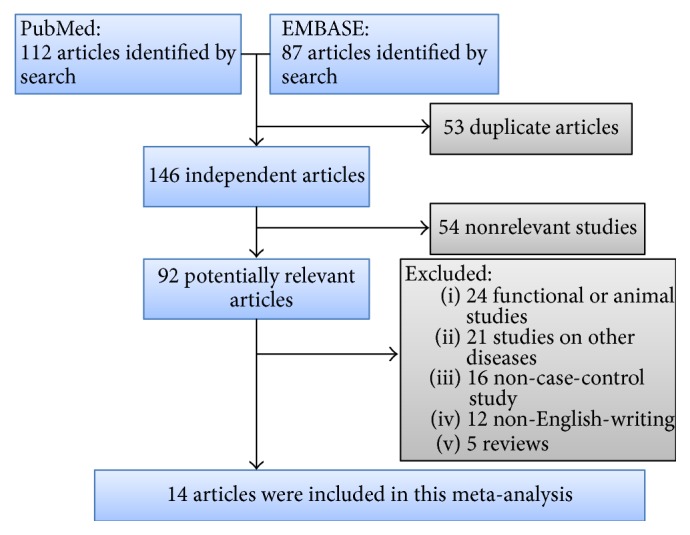
A schematic representation of the search strategy and selection process.

The search and selection process of included studies was presented in Figure 1. According to our searching strategy, 199 potentially relevant studies were retrieved initially; after screening, 14 studies and 30 extracted SNP outcomes met the inclusion criteria and were used for the meta-analysis. The general characteristics of the included studies were summarized in Table 1. Genotypic distribution was in agreement with HWE in all studies. The detailed information of the corresponding pooled odds ratios and *P* values of each SNP were presented in Table 2. The NOS results showed that the methodological quality was generally good (data not shown).

### 3.2. The Effect of p.Arg223Gln on T2DM

**Figure 2:**
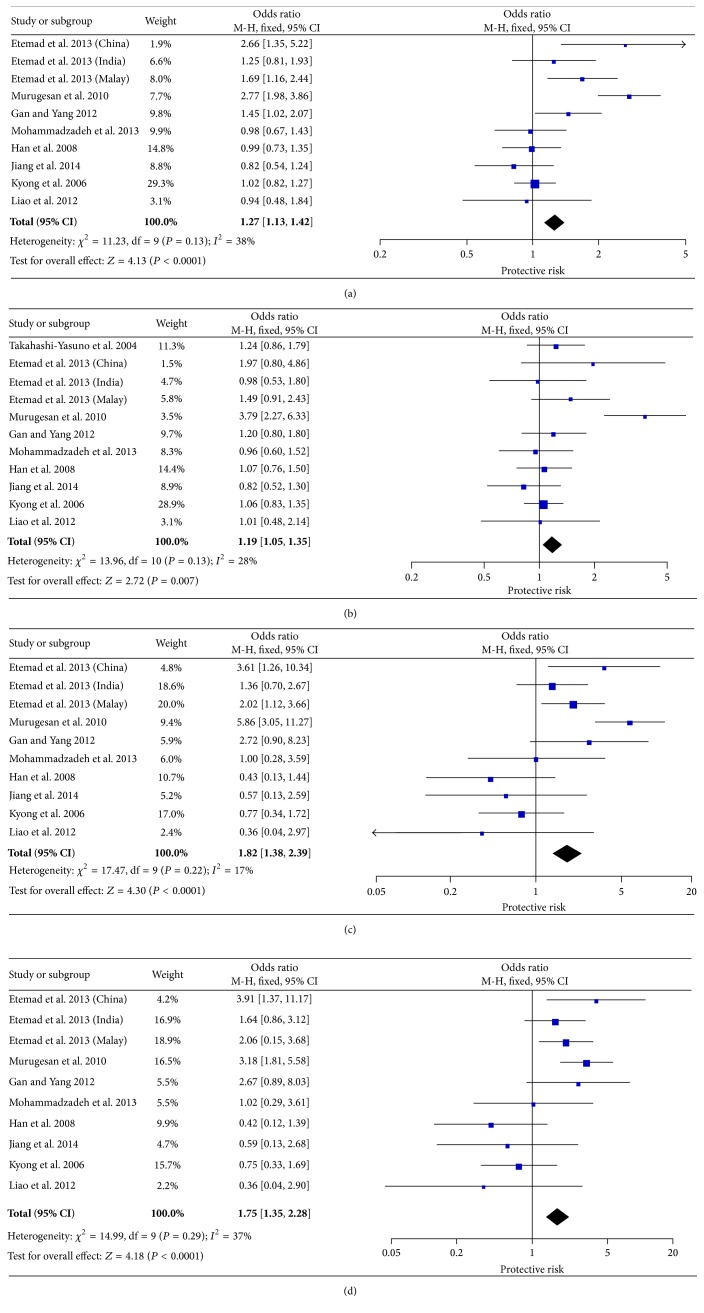
Forest plots of the association of* LEPR* p.R223Q with T2DM. The size of the box is proportional to the weight of the study, horizontal lines indicate 95% CI, and a diamond indicates the summary OR with its corresponding 95% CI. (a) Allele model, (b) dominant model, (c) homozygote model, and (d) recessive model; LEPR: leptin receptor gene; T2DM: type 2 diabetes mellitus; OR: odds ratio; CI: confidence interval.

Eleven studies, containing 3649 T2DM cases and 2381 controls, were eligible for pooling of genetic effects of p.R223Q on T2DM. The allele model (R versus Q) yielded a pooled OR of 1.27 (95% CI: 1.13–1.42) with minimal heterogeneity (*P*_Q_ = 0.13, *I* ^2^ = 38%). Our result demonstrated a positive correlation between R allele of p.R223Q and T2DM risk. Significant association was also observed under other genetic models (dominant: OR = 1.19, 95% CI 1.05–1.35; homozygote: OR = 1.82, 95% CI 1.38–2.39; and recessive: OR = 1.75, 95% CI 1.35–2.28, resp.) (Figure 2).

### 3.3. The Effect of p.R109K on T2DM

**Figure 3:**
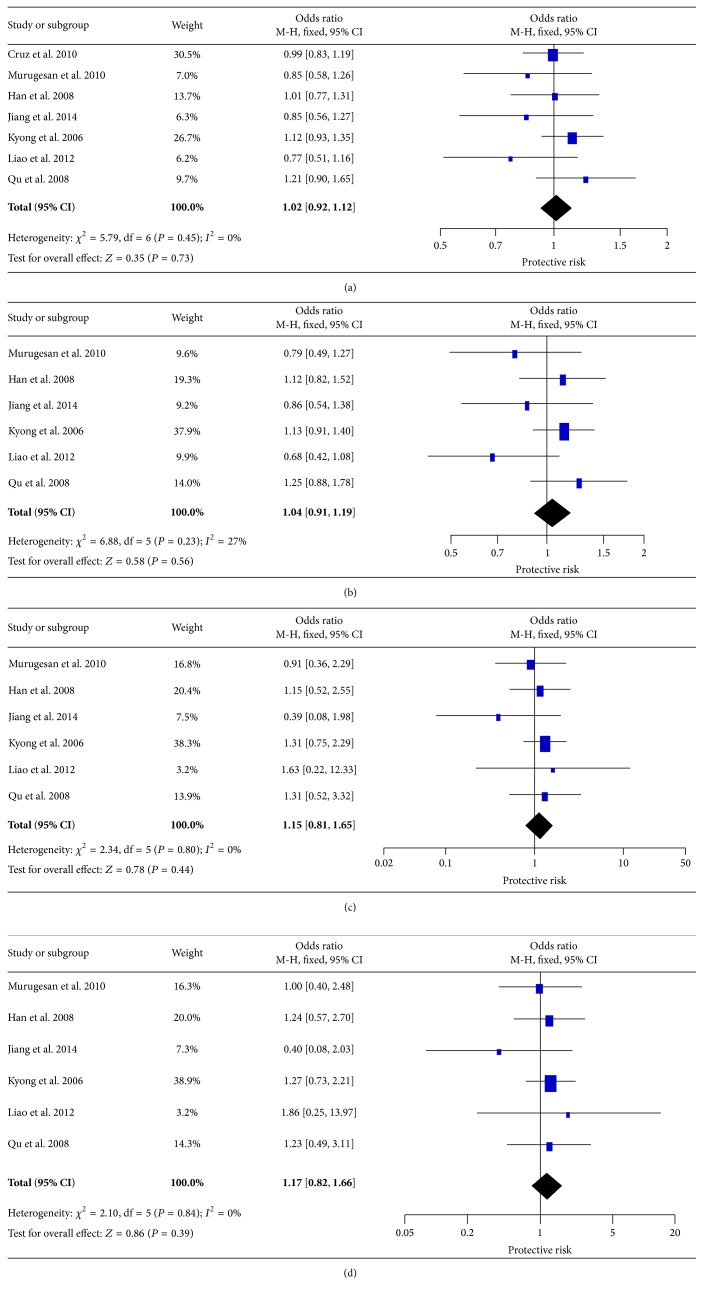
Forest plots of the association of* LEPR* p.R109K with T2DM. (a) Allele model, (b) dominant model, (c) homozygote model, and (d) recessive model.

For p.R109K variant, seven studies containing 3536 cases and 2268 controls were included. The pooled analysis showed that R109K had no significant association with T2DM susceptibility for all genetic models: allele (OR = 1.02, 95% CI 0.92–1.12), dominant model (OR = 1.04, 95% CI 0.91–1.19), homozygote model (OR = 1.15, 95% CI 0.81–1.65), and recessive model (OR = 1.17, 95% CI 0.82–1.66) (Figure 3). The ORs for all genetic effects were homogeneous across all studies (*I* ^2^ = 0%) except mild heterogeneity in the dominant model (*I* ^2^ = 27%).

### 3.4. The Effect of p.K656N on T2DM

**Figure 4:**
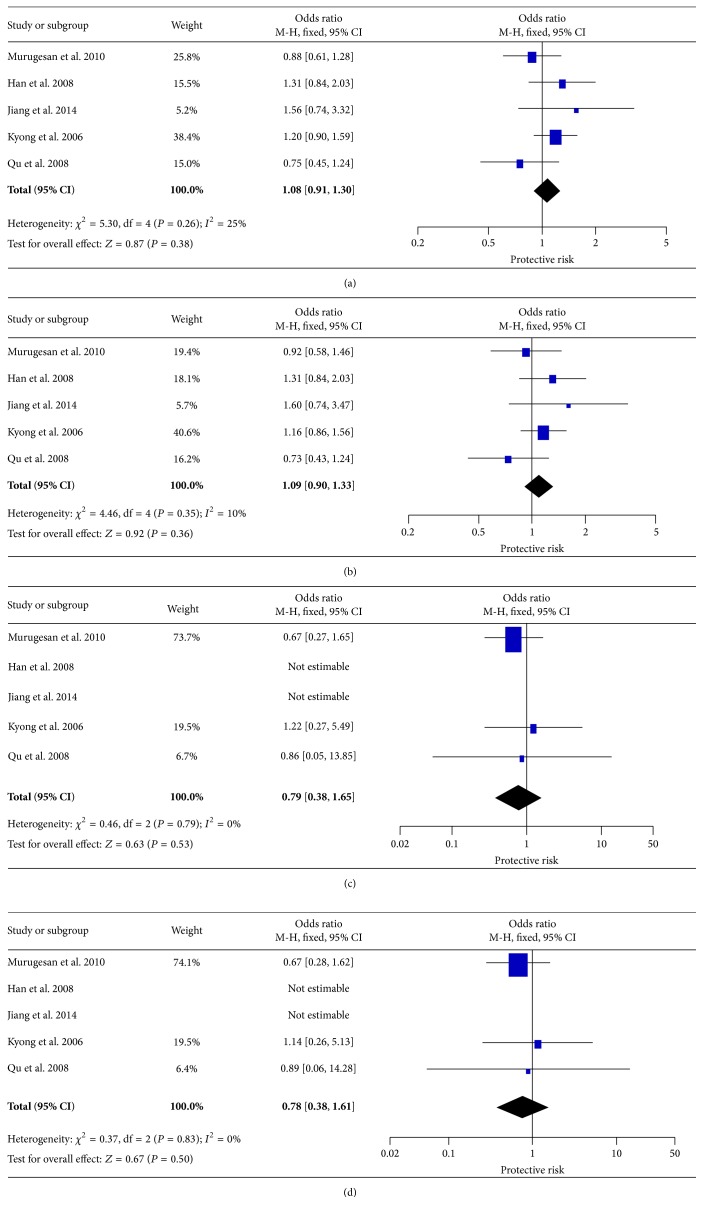
Forest plots of the association of* LEPR* p.K656N with T2DM. (a) Allele model, (b) dominant model, (c) homozygote model, and (d) recessive model.

The association between p.K656N and T2DM has been examined in five studies, including 2018 cases and 1641 controls. Overall, no significant association was observed in any genetic models. The genetic effects were homogeneous across studies (*I* ^2^ ≤ 25%, Figure 4).

### 3.5. Other Loci

**Figure 5:**
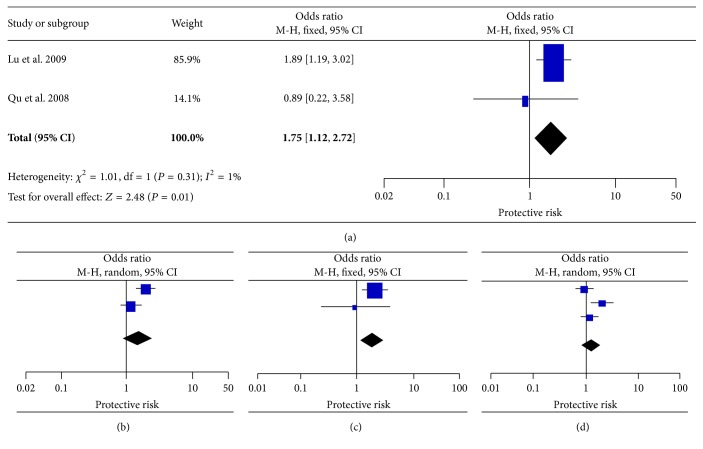
Forest plots of the association of* LEPR* p.P1019P with T2DM. (a) Recessive model, (b) allele model, (c) homozygote model, and (d) dominant model (study by Takahashi-Yasuno et al. only provides dominant data for this SNP).

**Figure 6:**
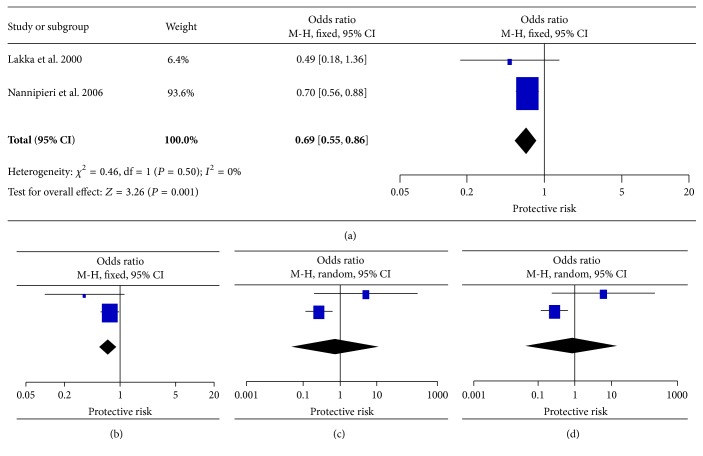
Forest plots of the association of* LEPR* 3′UTR with T2DM. (a) Allele model, (b) dominant model, (c) homozygote model, and (d) recessive model.

Three studies were carried out to assess the association of p.P1019P with susceptibility to T2DM, involving 753 cases and 767 controls. Only dominant genotype data can be extracted from the original study by Takahashi-Yasuno et al. [20]. Overall, the recessive and homozygote model showed homogeneity and the fixed effects pooled ORs were found to be significant (OR = 1.75, 95% CI = 1.12–2.72, and OR = 1.86, 95% CI = 1.14–3.02, resp.). The allele and dominant model yielded a strong heterogeneity between studies (*I* ^2^ ≥ 67%; *P* = 0.001), and the random effect model was therefore used but no significant association was observed (OR = 1.49, 95% CI = 0.89–2.50, and OR = 1.28, 95% CI = 0.84–1.96, resp.) (Figure 5). Regarding the association of 3′UTR ins/del, variable results were observed across different models, whose significant association was detected between 3′UTR ins/del and T2DM under allele and dominant models with no evidence of heterogeneity, but lack of significant association in other genetic models. Moreover, the ORs yielded strong heterogeneity among the studies (*I* ^2^ = 66% and 69% in homozygote and recessive model, resp.) (Figure 6). Two variants of rs1805134 (p.S343S) and rs22283014 (p.N567N) were reported by only one article by Kyong et al. with 775 cases and 688 healthy controls [17]. This study found no significant association between these two polymorphisms and risk of T2DM.

### 3.6. Heterogeneity and Publication Bias

**Figure 7:**
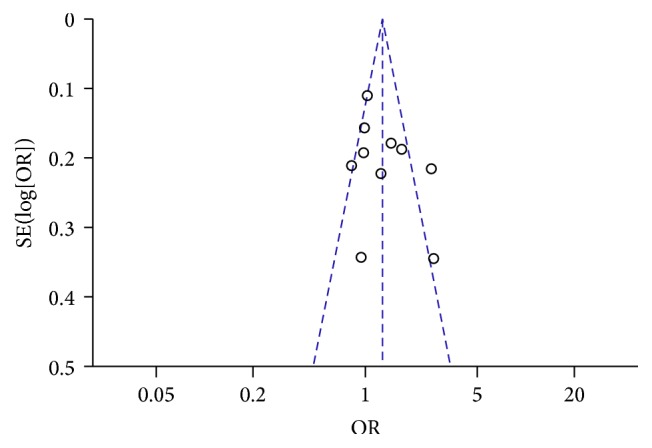
Funnel plot of studies conducted on the association between rs1137101 (R223Q) and T2DM risk (G>A).

In order to examine the influence of each study set to the pooled ORs, sensitivity analysis was performed by omitting one study in each time. For the variants, rs1137101 (p.R223Q), rs1137100 (p.R109K), and rs8179183 (p.K656N), the ORs were not significantly influenced by individual data, indicating that our results were statistically stable and robust. Metaregression was performed to detect the source of heterogeneity for the significant finding of rs1137101; the results indicated that sample size (*P* = 0.015) and source of control (*P* = 0.029) contributed heterogeneity, while ethnicity (*P* = 0.648) and publication year (*P* = 0.429) did not. Regarding rs62589000 (p.P1019P), the sensitivity analysis showed that the data from Lu et al. apparently influenced the overall results; therefore, the pooled ORs need be taken into account [15]. For 3′UTR ins/del, only two studies were performed in Finland and Mexican populations; among the two included articles, the results in one study performed by Nannipieri et al. were overweight and significantly influenced the overall results [23]. Publication bias was investigated by funnel plot and Egger's test; for all SNPs, funnel plot shapes did not reveal any evidence of obvious asymmetry (Figure 7). Egger's test also suggested no publication bias (*P* = 0.524 for rs1137101).

## 4. Discussion

In recent years, numerous studies have been conducted to evaluate the genetic influence of leptin to T2DM susceptibility. While the reports in different ethnic groups often yielded contradictory results, the inconsistencies could be due to the lack of power in each individual study with limited sample size as well as the heterogeneous data and methods. To confirm the association of* LEPR* and T2DM, we, for the first time, conducted a systematic review and meta-analysis to examine the associations of* LEPR* polymorphisms with T2DM risk. For this meta-analysis, five of the most commonly investigated* LEPR* SNPs were analyzed. Among them, p.R223Q was found to be significantly associated with T2DM, which remained significant even after sensitivity analysis. Genetic effect with allele R showed a positive association with T2DM from 1.19- to 1.82-fold under different genetic models. This result provides robust evidence for the genetic impact of leptin on diabetes, which will lead to further biological function investigation.

It is well known that obesity is a major link to T2DM, especially characterized by insulin resistance [24]. As leptin has long been linked with obesity, recent studies have depicted the role of leptin in T2DM and insulin resistance. Leptin can facilitate glucose utilization and improve insulin sensitivity, which has been implicated in the development of diabetes. Meanwhile, lower leptin expression in the adipose tissue and serum leptin levels were observed in T2DM patients [25]. The* LEPR* p.R223Q (G>A polymorphism, rs1137101) leads to an amino acid alteration from Arg to Gln located in the regulatory domain of the LEPR protein. Study by Murugesan et al. found that the levels of leptin, insulin, and body mass index (BMI) were significantly increased with homozygous and heterozygous variants of p.R223Q and showed significant difference between cases and controls [19]. These results were consistent with findings of Yiannakouris et al. suggesting that the p.R223Q polymorphism is associated with obesity and predicts a small percentage of body weight and body composition variability in a genetically homogeneous population [26]. Regarding this SNP, majority of studies were conducted in Asian populations; this is because some studies in other ancestries were excluded during the process of narrowing the studies; additionally, the MAF of this polymorphism is similar across different populations. Therefore, this significant association reported in the present study is more likely to be predictive of diabetes in overall population.

For other two functional variants, p.R109Q and p.K656N, no significant associations were found with T2DM under any genetic models, implying that these two variants might not contribute to the risk of T2DM. With regard to p.P1019P and 3′UTR ins/del, significant association was identified with T2DM, but the results were largely influenced by particular article and a limited number of studies were available. Therefore, these associations should be carefully interpreted, and further examinations in larger cohorts are warranted.

There are a number of limitations in our current meta-analysis. Firstly, only 14 available studies were enrolled. The number is not enough for every variant in the meta-analysis, such as p.P1019P and 3′UTR ins/del. Secondly, the small sample size conferred limited statistical power for exploring real association, especially for the subgroup analysis. Thirdly, as a multifactorial disease, a more precise analysis on T2DM-related factors should be conducted, such as BMI, age, and environmental exposure; however, some studies did not provide the detailed information.

To the best of our knowledge, this is the first meta-analysis to investigate the association of* LEPR* gene with T2DM. Our results suggested that the polymorphism rs1137101 (p.R223Q) has strong association with T2DM susceptibility. More studies in larger cohort and functional analyses of* LEPR* are required to reinforce the results and elucidate its biological roles in diabetes.

## Figures and Tables

**Table 1:** General characteristics of the studies included in this meta-analysis.

SNP	First author	Ethnicity	Case	Control	Age (mean ± SD)		Genotype frequency		BMI (kg/m^2^)		MAF
					Case	Control	Case	Control	Case	Control	
R223Q rs1137101	Jiang [13]	Chinese	369	176	68.0 ± 6.4^*∗*^	67.1 ± 7.1	4/65/273	3/33/117	25.0 ± 4.0^*∗*^	23.1 ± 3.3^*∗*^	0.42
	Mohammadzadeh [16]	Iranian	144	147	54.3 ± 8.9	52.5 ± 7.3	5/59/80	5/62/80	27.6 ± 4.5	28.0 ± 4.0	
	Gan [11]	Chinese	301	172	52.7 ± 10.7^*∗*^	52.8 ± 8.0	18/83/200	4/47/121	24.8 ± 2.3^*∗*^	24.8 ± 1.7^*∗*^	
	Murugesan [19]	Indian	150	150	NA	NA	53/67/30	22/55/73	26.2 ± 3.6^*∗*^	24.1 ± 3.4^*∗*^	
	Etemad [10]	Malaysian	145	133	NA	NA	42/17/86	22/20/91	NA	NA	
	Etemad [10]	Chinese	49	71	NA	NA	13/0/36	6/5/60	NA	NA	
	Etemad [10]	Indian	90	77	NA	NA	37/7/46	23/15/39	NA	NA	
	Han [12]	Korean	407	345	59.7 ± 9.62	64.5 ± 3.4	4/92/311	8/69/266	24.8 ± 3.1	24.0 ± 3.2	
	Takahashi-Yasuno [20]	Japanese	220	377	NA	NA	66/154^†^	97/279^†^	NA	NA	
	Liao [14]	Taiwanese	999	45	NA	NA	8/194/796	1/8/36	NA	NA	
	Kyong [17]	Korean	775	688	58.9 ± 10.5	64.2 ± 4.2	11/177/578	13/148/523	24.4 ± 2.9	23.6 ± 3.1	

R109K rs1137100	Jiang [13]	Chinese	369	176	68.0 ± 6.4^*∗*^	67.1 ± 7.1	3/81/184	3/35/72	25.0 ± 4.0	23.1 ± 3.3	0.32
	Murugesan [19]	Indian	150	150	NA	NA	10/40/100	10/48/91	25.7 ± 4.8^*∗*^	24.7 ± 3.6^*∗*^	
	Cruz [21]	Mexican	519	547	53.4 ± 7.4	43.6 ± 6.6	NA	NA	29.3 ± 4.8	27.5 ± 3.6	
	Kyong [17]	Korean	775	688	58.9 ± 10.5	64.2 ± 4.2	31/238/496	22/200/461	24.4 ± 2.9	23.6 ± 3.1	
	Qu [18]	Chinese	317	282	49.3 ± 13.7	45.2 ± 5.7	11/93/213	8/71/203	25.5 ± 4.1	23.8 ± 2.9	
	Liao [14]	Taiwanese	999	80	NA	NA	23/265/705	1/29/50	NA	NA	
	Han [12]	Korean	407	345	59.7 ± 9.62	64.5 ± 3.4	16/122/269	11/97/235	24.8 ± 3.1	24.0 ± 3.2	

K656N rs8179183	Jiang [13]	Chinese	369	176	68.0 ± 6.4^*∗*^	67.1 ± 7.1	0/34/226	0/9/96	25.0 ± 4.0	23.1 ± 3.3	0.14
	Murugesan [19]	Indian	150	150	NA	NA	9/52/89	13/51/86	25.8 ± 4.3^*∗*^	24.7 ± 4.0^*∗*^	
	Kyong [17]	Korean	775	688	58.9 ± 10.5	64.2 ± 4.2	4/111/650	3/84/596	24.4 ± 2.9	23.6 ± 3.1	
	Qu [18]	Chinese	317	282	49.3 ± 13.7	45.2 ± 5.7	1/27/289	1/32/249	25.5 ± 4.1	23.8 ± 2.9	
	Han [12]	Korean	407	345	59.7 ± 9.62	64.5 ± 3.4	0/57/350	0/38/305	24.8 ± 3.1	24.0 ± 3.2	

P1019P rs62589000	Lu [15]	Chinese	216	108	NA	NA	132/33/51	49/17/42	27.0 ± 4.1^*∗*^	22.3 ± 2.2	0.31
	Takahashi-Yasuno [20]	Japanese	220	377	NA	NA	57/163^†^	102/274^†^	NA	NA	
	Qu [18]	Chinese	317	282	49.3 ± 13.7	45.2 ± 5.7	4/69/244	4/53/225	25.5 ± 4.1	23.8 ± 2.9	

3′UTR Ins/Del	Lakka [22]	Finland	41	81	52.9 ± 6.0	52.9 ± 6.4	1/3/37	0/19/62	NA	NA	NA
	Nannipieri [23]	Mexican	503	609	NA	NA	7/124/372	29/167/413	NA	NA	

S343S rs1805134	Kyong [17]	Korean	775	688	58.9 ± 10.5	64.2 ± 4.2	4/103/659	3/63/616	24.4 ± 2.9	23.6 ± 3.1	0.25

N567N rs2228301	Kyong [17]	Korean	775	688	58.9 ± 10.5	64.2 ± 4.2	0/3/769	0/7/676	24.4 ± 2.9	23.6 ± 3.1	NA

BMI: body mass index; NA: not available; MAF: minor allele frequency.

For allele and genotype frequency, the rare allele was defined as reference.

^*∗*^Data was calculated manually by combining subgroup data.

^†^Only genotype counts in dominant model can be extracted from the original study.

**Table 2:** Pooled analyses on the association of *LEPR* gene polymorphisms with T2DM.

Polymorphism	Cases/controls	Genetic model	OR (95% CI)	*P* value	*I* ^2^ (%)	*P*_*Q*_
p.Arg223Gln (R223Q)	3649/2381	Allele	1.27 [1.13, 1.42]	<0.0001	38	0.13
		Dominant	1.19 [1.05, 1.35]	0.007	28	0.13
		Homozygote	1.82 [1.38, 2.39]	<0.0001	17	0.22
		Recessive	1.75 [1.35, 2.28]	<0.0001	37	0.29

p.Arg109Lys (R109K)	3536/2268	Allele	1.02 [0.92, 1.12]	0.73	0	0.45
		Dominant	1.02 [0.86, 1.20]	0.86	27	0.23
		Homozygote	1.15 [0.81, 1.65]	0.44	0	0.8
		Recessive	1.17 [0.82, 1.66]	0.39	0	0.84

p.Lys656Asn (K656N)	2018/1641	Allele	1.08 [0.91, 1.30]	0.38	20	0.26
		Dominant	1.09 [0.90, 1.33]	0.36	10	0.35
		Homozygote	0.79 [0.38, 1.65]	0.53	0	0.79
		Recessive	0.78 [0.38, 1.61]	0.5	0	0.83

p.Pro1019Pro (P1019P)	753/767	Allele	1.49 [0.89, 2.50]	0.13	78	0.03
		Dominant	1.28 [0.84, 1.96]	0.25	67	0.05
		Homozygote	1.86 [1.14, 3.02]	0.01	10	0.29
		Recessive	1.75 [1.12, 2.72]	0.01	1	0.31

3′UTR ins/del	544/690	Allele	0.69 [0.55, 0.86]	0.001	0	0.5
		Dominant	0.71 [0.55, 0.92]	0.008	34	0.22
		Homozygote	0.75 [0.05, 11.7]	0.84	66	0.08
		Recessive	0.35 [0.16, 0.76]	0.92	69	0.07

## References

[1] Shaw J. E., Sicree R. A., Zimmet P. Z. (2010). Global estimates of the prevalence of diabetes for 2010 and 2030. *Diabetes Research and Clinical Practice*.

[2] Waugh N. R., Shyangdan D., Taylor-Phillips S., Suri G., Hall B. (2013). Screening for type 2 diabetes: a short report for the National Screening Committee. *Health Technology Assessment*.

[3] Rudkowska I., Pérusse L. (2012). Individualized weight management: what can be learned from nutrigenomics and nutrigenetics?. *Progress in Molecular Biology and Translational Science*.

[4] Banerjee M., Saxena M. (2014). Genetic polymorphisms of cytokine genes in type 2 diabetes mellitus. *World Journal of Diabetes*.

[5] Klok M. D., Jakobsdottir S., Drent M. L. (2007). The role of leptin and ghrelin in the regulation of food intake and body weight in humans: a review. *Obesity Reviews*.

[6] Brennan A. M., Mantzoros C. S. (2006). Drug Insight: the role of leptin in human physiology and pathophysiology—emerging clinical applications. *Nature Clinical Practice Endocrinology and Metabolism*.

[7] Pan H., Guo J., Su Z. (2014). Advances in understanding the interrelations between leptin resistance and obesity. *Physiology and Behavior*.

[8] Ren J. (2004). Leptin and hyperleptinemia—from friend to foe for cardiovascular function. *Journal of Endocrinology*.

[9] Jéquier E. (2002). Leptin signaling, adiposity, and energy balance. *Annals of the New York Academy of Sciences*.

[10] Etemad A., Ramachandran V., Pishva S. R. (2013). Analysis of Gln223Agr polymorphism of Leptin Receptor gene in type II diabetic mellitus subjects among Malaysians. *International Journal of Molecular Sciences*.

[11] Gan R.-T., Yang S.-S. (2012). The 223A>G polymorphism of the leptin receptor gene is associated with macroangiopathy in type 2 diabetes mellitus. *Molecular Biology Reports*.

[12] Han H. R., Ryu H.-J., Cha H. S. (2008). Genetic variations in the leptin and leptin receptor genes are associated with type 2 diabetes mellitus and metabolic traits in the Korean female population. *Clinical Genetics*.

[13] Jiang B., Liu Y., Liu Y., Fang F., Wang X., Li B. (2014). Association of four insulin resistance genes with type 2 diabetes mellitus and hypertension in the Chinese Han population. *Molecular Biology Reports*.

[14] Liao W.-L., Chen C.-C., Chang C.-T. (2012). Gene polymorphisms of adiponectin and leptin receptor are associated with early onset of type 2 diabetes mellitus in the Taiwanese population. *International Journal of Obesity*.

[15] Lu H., Sun J., Sun L., Shu X., Xu Y., Xie D. (2009). Polymorphism of human leptin receptor gene is associated with type 2 diabetic patients complicated with non-alcoholic fatty liver disease in China. *Journal of Gastroenterology and Hepatology*.

[16] Mohammadzadeh G., Nikzamir A., Mohammadi J., Pourdashti S., Shabazian H., Latifi S.-M. (2013). Association of the 223A/G LEPR polymorphism with serum leptin levels in Iranian subjects with type 2 diabetes. *Archives of Iranian Medicine*.

[17] Kyong S. P., Hyoung D. S., Byung L. P. (2006). Polymorphisms in the leptin receptor (LEPR)—putative association with obesity and T2DM. *Journal of Human Genetics*.

[18] Qu Y., Yang Z., Jin F. (2008). The haplotype identified in LEPR gene is associated with type 2 diabetes mellitus in Northern Chinese. *Diabetes Research and Clinical Practice*.

[19] Murugesan D., Arunachalam T., Ramamurthy V., Subramanian S. (2010). Association of polymorphisms in leptin receptor gene with obesity and type 2 diabetes in the local population of Coimbatore. *Indian Journal of Human Genetics*.

[20] Takahashi-Yasuno A., Masuzaki H., Miyawaki T. (2004). Association of Ob-R gene polymorphism and insulin resistance in Japanese men. *Metabolism: Clinical and Experimental*.

[21] Cruz M., Valladares-Salgado A., Garcia-Mena J. (2010). Candidate gene association study conditioning on individual ancestry in patients with type 2 diabetes and metabolic syndrome from Mexico city. *Diabetes/Metabolism Research and Reviews*.

[22] Lakka H.-M., Oksanen L., Tuomainen T.-P., Kontula K., Salonen J. T. (2000). The common pentanucleotide polymorphism of the 3'-untranslated region of the leptin receptor gene is associated with serum insulin levels and the risk of type 2 diabetes in non-diabetic men: a prospective case-control study. *Journal of Internal Medicine*.

[23] Nannipieri M., Posadas R., Bonotti A. (2006). Polymorphism of the 3′-untranslated region of the leptin receptor gene, but not the adiponectin SNP45 polymorphism, predicts type 2 diabetes: a population-based study. *Diabetes Care*.

[24] Milan G., Granzotto M., Scarda A. (2002). Resistin and adiponectin expression in visceral fat of obese rats: effect of weight loss. *Obesity Research*.

[25] Dardeno T. A., Chou S. H., Moon H.-S., Chamberland J. P., Fiorenza C. G., Mantzoros C. S. (2010). Leptin in human physiology and therapeutics. *Frontiers in Neuroendocrinology*.

[26] Yiannakouris N., Yannakoulia M., Melistas L., Chan J. L., Klimis-Zacas D., Mantzoros C. S. (2001). The Q223R polymorphism of the leptin receptor gene is significantly associated with obesity and predicts a small percentage of body weight and body composition variability. *Journal of Clinical Endocrinology and Metabolism*.

